# Understanding Karma Police: The Perceived Plausibility of Noun Compounds as Predicted by Distributional Models of Semantic Representation

**DOI:** 10.1371/journal.pone.0163200

**Published:** 2016-10-12

**Authors:** Fritz Günther, Marco Marelli

**Affiliations:** 1 Department of Psychology, University of Tübingen, Tübingen, Germany; 2 Department of Experimental Psychology, Ghent University, Ghent, Belgium; University of Akron, UNITED STATES

## Abstract

Noun compounds, consisting of two nouns (the *head* and the *modifier*) that are combined into a single concept, differ in terms of their plausibility: *school bus* is a more plausible compound than *saddle olive*. The present study investigates which factors influence the plausibility of attested and novel noun compounds. Distributional Semantic Models (DSMs) are used to obtain formal (vector) representations of word meanings, and compositional methods in DSMs are employed to obtain such representations for noun compounds. From these representations, different plausibility measures are computed. Three of those measures contribute in predicting the plausibility of noun compounds: The relatedness between the meaning of the head noun and the compound (Head Proximity), the relatedness between the meaning of modifier noun and the compound (Modifier Proximity), and the similarity between the head noun and the modifier noun (Constituent Similarity). We find non-linear interactions between Head Proximity and Modifier Proximity, as well as between Modifier Proximity and Constituent Similarity. Furthermore, Constituent Similarity interacts non-linearly with the familiarity with the compound. These results suggest that a compound is perceived as more plausible if it can be categorized as an instance of the category denoted by the head noun, if the contribution of the modifier to the compound meaning is clear but not redundant, and if the constituents are sufficiently similar in cases where this contribution is not clear. Furthermore, compounds are perceived to be more plausible if they are more familiar, but mostly for cases where the relation between the constituents is less clear.

## Introduction

A central feature of language is the possibility for speakers to use words from their finite vocabulary and combine them in new ways to express novel meanings. This property enables speakers to express meanings that may never have been expressed before, by using word combinations, such as sentences, phrases, or other complex expressions.

*Noun compounds* (also referred to as *nominal compounds*), such as *apple pie*, *mountain top*, *rock music* or *beach party* are one instance of such expressions (for a differentiation between phrases and compounds, see [1], [2], and the next section of the present article for an overview). Some compounds, such as *school bus*, are frequently used, and some are highly lexicalized [3] [4], such as *airport* or *soap opera*. However, it is also possible to create new compounds that a listener maybe never has encountered before [5], and novel compounds can usually be generated and understood without problems. Of these noun compounds, however, some might be quite easy to interpret, such as *moon colonist*, while it might be harder, but still possible, to interpret a compound such as Radiohead’s *karma police* [6]. For others, such as *saddle olive*, a sensible interpretation can be almost impossible.

Given these examples, it is obvious that noun compounds differ in terms of plausibility. However, although a lot of work has been done on how compounds are formed and interpreted, it is still quite unclear which factors actually influence whether humans perceive a compound to be plausible or not. Indeed, this aspect is not often addressed in morphological theories, that do rarely consider the semantics-pragmatics interface and cognitive aspects with regards to compound interpretation. However, a morphologically complex word can be perfectly legal, but still be considered meaningless by native speakers (for example, see the discussion in [7] on derivation). Plausibility then becomes a central topic of research in cognitively-oriented studies on compound comprehension, which are mostly interested in compound words as a window on the human ability to combine existing concepts in novel and creative ways, allowing one to explore new thoughts and imagine new possibilities. This is most evident from proposals in the conceptual combination domain [8], [9], [10], [11], [12], where plausibility is considered to be one of the major variables that theories of conceptual combination have to explain [8], [10]. As a result, compound plausibility is a crucial variable to investigate for models concerned with how we are able to understand compound meanings in a seamingly effortless manner.

In our study, we investigate which factors influence human judgements on the plausibility of (English) noun compounds. First, we discuss linguistic approaches to compounding as well as psychological models of conceptual combination as a theoretical background, and propose recent developments in the computational linguistic field of compositional distributional semantics as a methodological framework and a formalized, algorithmic implementation of these models. We then review previous findings and assumptions concerning the determinants of plausibility judgements, and present measures in compositional distributional semantics that capture and extend those findings.

### Noun compounds—Definition and Classification

Setting a rigorous and foolproof definition for what counts as a noun compound is a rather difficult issue, and to almost any definition criterion one can find examples that appear to be misclassified if the criterion is rigorously applied, see [1], [2]. For the purpose of the present study, we apply a rather broad definition (compare, for example, [13]): In the text that follows, we use the term “noun compound” to refer to a construction of two adjoined and inseparable nouns that denotes a single new concept [2], [14], and functions as a noun itself (in short, it is of the [N + N]_N_ type). This rather broad and agnostic view on compounds converges with the view held in the psychological literature of conceptual combination [15], [16], where it has to be explained for any compound, how the concept denoted by it (e. g.*flower pot*) is formed from the concepts denoted by its constituents (*flower* and *pot*).

Note that some theorists assume that the term “compound” should only be used when referring to idiomatic and therefore necessarily non-compositional [N + N]_N_ constructions [17], [14]. However, since our present analysis relies on compositionally derived representations of compound meanings, such a definition is incompatible with our approach. Therefore, if one applies the idiomatic (or any other non-compositional) definition of compounds, then the present study should be seen as dealing with phrases of the [N + N]_N_ type (see, for example, [1], [4], [18], for further discussions on how to distinguish phrases from compounds).

As mentioned in the previous paragraph, noun compounds consist of two elements, called *constituents*. The *head* typically denotes the semantic category a compound belongs to [19]; for example, a *swordfish* is a kind of fish and not a kind of sword, and *fish* is the head constituent. The role of the other constituent (*sword*) is to modify and specify this head, therefore it is referred to as the *modifier*. Due to this specification, the entities referred to by the compound (all *swordfish*) are a subset of the entities referred to by the head noun (all *fish*), which constitutes a hyponymy relation, as incorporated in the IS A Condition proposed in [20]: In a compound [X Y]_Z_ (i.e., the compound Z with the constituents X and Y), Z ’IS A’ Y. For English, the *right-hand head rule* [21] states that the head of a noun compound always is the final (i.e., the right-hand side) constituent. However, this is not the case for all languages: In Italian, a swordfish is referred to as *pesce spada* (*fish-sword*). Hence, due to issues such as headedness, compounds are considered to be inherently asymmetrical in structure (except for maybe coordinates, see below; [22], [23].)

On basis of the role these constituents play, compounds can be classified into different categories (e.g., [24], [18], [25]). The classification in [25] postulates three major categories: In coordinate compounds, such as *singer-songwriter* or *prince-bishop*, the denoted concept is of the “the first constituents but also the second constituent” type. For example, a *prince-bishop* is a person who at the same time holds the spiritual office of a bishop, but also the secular office of a prince; he is simultaneously a bishop as well as a prince. In subordinate compounds, such as *taxi driver* or *train station*, there is a head-complement relation between the two constituents. Hence, one of the constituents licenses an argument, and the other constituent is taken as an argument to fill that role. In attributive compounds, such as *snail mail* or *key word* or *ghost writer*, a feature of the modifier is taken to specify a feature of the head noun, as in the *swordfish* example above. As argued in [26], attributive compounds are the most common type of compounds in many languages, and are to be found when the constituents are (structurally and semantically) too dissimilar to be interpreted as coordinates, and lack the argument structure to be interpreted as subordinates. Compounds in all three classes can be subdivided into *endocentric* compounds, which are an actual member of the category denoted by the head noun and hence are hyponyms of the head (such as *apple pie*, *state police*, or *bee hive*), and *exocentric* compounds, where this is, strictly speaking, not the case (take, for example, *metalhead*, *freelancer* or *treadmill*; but [27]). Hence, a *metalhead* is not a head, but a person who is very much into metal music.

In the present study, we will try to formulate a general framework for the plausibility of noun compounds. To this end, we work under the hypothesis that humans do not a priori distinguish between the different categories of noun compounds in order to apply a specifically tailored plausibility judgement mechanism for the specific compound class.

### The Plausibility of Noun Compounds

#### Terminology—Acceptability, Plausibility, Meaningfulness

In the literature, various terms are being used for the concept of plausibility [28], and the term *plausibility* is used to describe different concepts [28], [9].

[28], [29] use the term *plausibility* (while emphasizing the difficulties in defining it), and state that it is often defined operationally: Plausibility is obtained through human ratings of plausibility. They also point out the apparently synonymous usage of other terms, like *sensible* and *makes sense*. In another study [30], those ratings are referred to as judgements of *meaningfulness*, without further defining this term. This term was also used in [7] to describe the relative acceptability of affix-word combinations. Conversely, [31] used the term *semantic deviance* to describe expressions that cannot be interpreted in normal communicative contexts and are therefore implausible.

In the model in [9], plausibility is given if a compound describes something that the listener can refer it to (for example, the compound *eucalyptus bear* is plausible if you know about the existence and eating habits of koalas). In this model, the *acceptability* of an interpretation for a compound is then a function of (amongst others) its plausibility.

For the remainder of this paper, we will assume as a working hypothesis that plausibility, acceptability, meaningfulness, and semantic deviance subtend the same latent variable. We therefore assume that these terms can be used interchangeably for our purposes. For the remainder of this article, we will keep to the term *plausibility*.

#### Stages of Plausibility Judgements

As pointed out in [29], although plausibility ratings have often been used to explain various cognitive phenomena (for example in the areas of reasoning, memory, and problem solving), it received little attention as a variable of interest in itself.

To overcome this gap, these authors proposed the Plausibility Analysis Model (PAM) [32], [28], [29]. The main focus of this model are plausibility judgements for whole scenarios consisting of multiple sentences, such as *The bottle fell off the shelf. The bottle smashed*. However, it also provides a useful theoretical background for plausibility judgements on simpler expressions, such as noun compounds.

In this model, plausibility judgements are the result of two stages: A *comprehension stage* and an *assessment stage*. During the comprehension stage, a mental representation for the input (i.e., the compound) is obtained. The plausibility of this representation is then evaluated in the assessment stage. The main assumption in PAM is that it is assessed whether the obtained representation is in line with prior knowledge. Especially, it is examined whether the concepts that are part of the mental representation are coherent.

### The Comprehension of Noun Compounds

#### Linguistic Approaches—The Problem of Interpretation

In the linguistic literature, the issue of how meanings are assigned to compounds, and to what extent these interpretations of a compound’s meaning can be predicted, for example from its constituents, is referred to as the *problem of interpretation* [2], [33].

In his seminal generative approach to compounds, [34] advocates the idea that compounds are transformations of sentences [35], or noun-like versions of sentences that are stripped of some grammatical elements and re-arranged. Consider as an example a compound such as *stone wall*. For the purpose of illustration, we will start from the sentence *The wall is built out of stones*. One possible transformation of this sentence is the sequence *… wall built out of stones …* which can be used in a noun-like fashion (e.g., *The guardian continued his patrol on the wall built out of stones*). The compound *stone wall* then is a transformation of this sequence, and can be used instead of the sequence: *The guardian continued his patrol on the stone wall*. The basic idea of this approach is that these examples share the same deep structure from which they are generated. The meaning of the compound is then given by the deep structure from which it was generated. The relation between compounds and syntactic structures is particularly evident for head-initial compounds in Romance languages [36], in which prepositional compounds are also observed [37]. In Italian, for example, the same compound can be expressed through a head-initial structure (e.g., *cabina telefonica*, *phone booth*, lit. *booth telephone_adj_*) or a prepositional structure (e.g., *cabina del telefono*, lit. *booth of the telephone_noun_*).

On the other hand, according the lexicalist approach to compounding [20], [38], [39], it is assumed that the lexicon and the lexical semantics of the constituents carry the workload of compounding, not the underlying deep structure. Thus, the lexicalist approach assumes that the constituents of a compound determine its meaning, and not its construction (see also [5]). This is illustrated in the Variable R Condition proposed in [20]: In the primary compound [X Y]_Z_, the meaning of X fills any one of the feature slots of Y that can be appropriately filled by X.

The lexical semantic approach [39], [26] builds on and further specifies this point. According to Lieber [39], [26], the semantic representation of a morpheme (in this case, a constituent) consists of a semantic/grammatic skeleton that contains all its (semantic) features that are relevant to the syntax of a language. Examples in English are whether an entity is a concrete or an abstract noun, or whether it is static or dynamic. In addition to the skeleton, the representation also entails the semantic/pragmatic body, which includes other features of and knowledge about the constituent, for example that a dog has four legs and that it barks. The studies in [39], [26] then analyse compounding for the three classes of compounds [25] (we will focus on endocentric compounds here): For coordinate compounds such as *singer-songwriter*, that share a large amount of features, the skeleton and the body are assumed to be highly similar and therefore easily coindexed (*coindexation* in this context is to be understood as “identified as referring to the same entity”). They will also differ in some features, and those features can either be interpreted as being simultaneously true, as in the case of *singer-songwriter*, or mixed, as in the case of *blue-green*. For subordinate compounds such as *taxi driver* or *football player*, Lieber argues that the heads (*driver* and *player*) have free slots for arguments (specifying what is driven and what is played), and this role is filled by the modifiers. In most cases, such a process can work on the level of the semantic/grammatic skeletons alone. Finally, for attributive compounds such as *horror story* or *doghouse*, which are allegedly the most frequent and most productive in English [26], the case is somewhat different: Although their skeletons can be very similar (*dog* and *house* are both concrete objects), their bodies can differ quite substantially (a dog is animate, not human, has four legs and barks, while a house is not animate, and artefact, and has windows and a door).

In another approach, Jackendoff [40] proposes that interpreting the semantic structure of a compound relies on two factors: on the one hand, the head of the compound has to be identified, and on the other hand, the semantic relation between the constituents has to be determined. He identifies two main schemata for this semantic relation: One schema is the *argument schema*, where a compound [X Y] is an Y by/of/… Z. This schema is most prominently realized in subordinate compounds. Attributive compounds, however, can in most cases not be interpreted with this schema, and the relationship between the constituents—or, in other words, which features of the head are affected in which way by the modifier’s features—is not fixed and therefore free and potentially ambiguous, or promiscuous [40]: A *dog house* can be a house in which dogs live, or a house in the shape of a dog, or a strange house which consists of dogs as building blocks. Following Jackendoff, the *modifier schema* is applied in such cases: [X Y] is an Y such that some F is true for both X and Y. Interpreting the meaning of [X Y] then is identifying F, or, in other words, the specific relation between X and Y. Possible candidates for such a relation, which is argued not to be completely arbitrary but rather an element of a finite set of possible relations, include a LOCATION relation (Y is located at/in/on X, as for *mountain pass*), a SERVES AS relation (Y serves as X, as for *buffer state*), or a CAUSE relation (Y is caused by X, as for *knife wound*); for a more complete list of relations, see [40].

Taken together, the main idea of such lexical approaches is that both constituents are defined by a set of semantic features, which are combined, selected or changed in the compound generated from the constituents.

One commonality of many theories on compounding, including generative and lexicalist approaches, is the view that an important part of interpreting a compound’s meaning is to interpret the relation between its constituents, that is, to identify Allen’s [20] type R relation (e.g., [41], [5], [42], [40], [43], [26]). As an illustration, a *wind mill* is usually interpreted as a mill that is powered by wind, but other interpretations are also available given an appropriate context: for example, a *wind mill* could, in some other world, also be a mill that produces wind (compare *flour mill*). A major task of many of these theories is to identify possible relations between the constituents, and to classify given compounds with respect to these relations. For example, [43] postulates a set of nine different relations, which, amongst others, include a CAUSE relation (e.g., *air pressure, accident weather*), a HAVE relation (e.g., *city wall, picture book*), or a USE relation (e.g., *wind mill*).

#### Psychological Approaches—Conceptual Combination

In the psychological literature, the process of combining two concepts into a new one (as for adjective-noun compounds or noun-noun compounds) is referred to as *conceptual combination* (see [15], [16] for reviews on this topic).

Probably the first psychological model of conceptual combination is the Selective Modification Model [44], [11]. This model assumes concepts to be stored in memory as prototype schemata, which consist of a set of dimensions. Each of these dimensions includes a range of features (the dimension *colour*, for example, can include the features *red*, *blue* and *green*), and each of those features is weighted by a numerical value of “votes” (for the concept *sky*, the feature *blue* probably has the highest vote count on the dimension *colour*, soon followed by *grey*). Furthermore, the model also postulates a numerical diagnosticity value to be assigned to the dimensions: For the concept *sky*, the dimension *colour* most likely has a higher diagnosticity than the *smell* dimension, while the opposite should be the case for *perfume*.

However, the focus of the Selective Modification Model were adjective-noun combinations, and not noun compounds. An early model dealing with noun compounds is the Concept Specialization Model [45], [46], [47], which can be considered an extension of the Selective Modification Model [16]. This model assumes a similar representation of concepts, namely as prototype schemata with slots (i.e., dimensions) and fillers (i.e., values on these dimensions). When a head noun is combined with a modifier, the concept given by the head noun is then altered as a function of the modifier concept. More specifically, it is assumed that the modifier fills in specific slots of the head noun concept, which is a specialization of the head noun concept. The selection and filling of slots is guided by background knowledge. In the case of the compound *moon colonist*, the head noun *colonist* might for example have a slot for LOCATION and for AGENT. When this concept is combined with the modifier *moon*, the LOCATION slot is then filled with *moon*. That *moon* is more suitable as a LOCATION than an AGENT is determined by the listener’s background knowledge on the nature of colonisation (usually, this is a process of people settling on some land), and of the moon (which is an area that could in principle be settled on). As can be seen, these approaches resemble the core idea of lexicalist approaches to compound meanings [20], [39], [26], which assume that one constituent of the compound (the modifier) specifies certain features of the other constituent (the head).

Over the following decades, several additional models on conceptual combination have been proposed [48], [49], [42], [12], [50], [9], [51]. As argued and illustrated in [16], those can be seen as extensions or specifications of the Selective Modification Model and the Concept Specialization model. Although they differ in their scope and theoretical assumptions on how the process of conceptual combination works, and how interpretations for compounds are obtained, they share the basic assumptions of concepts being represented as prototype schemata with dimensions. Furthermore, they assume that the combination process modifies the head noun’s values on these dimensions with respect to the modifier noun, which is an instantiation and specific implementation of identifying Allen’s (1978) Relation R.

Notably, the Competition Among Relations in Nominals (CARIN) model by Gagné [42], [52], [53] postulates that a crucial part of conceptual combination is to identify a thematic relation between the constituents of a compound (see also the present version of CARIN, the RICE model, for an updated formalization [54]). This approach is therefore very similar to linguistic theories that focus on relations between constituents to address the problem of interpretation ([5], [43], also see the respective paragraphs in the previous section). According to the CARIN model, relations are known from prior experience, and have to be filled in for a given compound that is encountered. Hence, the CARIN model assumes that a concept has slots for thematic relations that can link the concept to other concepts. The likelihood that a given relation is chosen for the interpretation of a given compound then depends on prior experience: For example, *river mill* will be most likely identified as a mill that is located nearby a river, since the modifier *river* if often used to establish a locative relation in compounds.

#### The Pragmatics of Conceptual Combination

While most psychological models of conceptual combination are focussed on compositional semantics (i.e., how the meaning of the compound is formed as a function of its constituents), the Constraint Model [9] employs pragmatical principles of communication. Central to this model is the assumption that the speaker and the listener in a communicative situation are cooperative [55]. This especially implies that the speaker tries to choose the best-fitting expression in order to transfer an intended meaning to the listener.

From this assumption, [9] derive three pragmatical constraints concerning the meaning of compounds: As stated earlier, *plausibility* indicates whether the compounds refers to something that the listener can be assumed to know. If the listener does not know about the concept of koalas (and especially their eating habits), a more detailed description of the concept than *eucalyptus bear* would be more adequate. *Diagnosticity* indicates whether the combined concept is best identified by the specific constituents of the compounds. We can assume diagnosticity to be quite high for *eucalyptus bear*, which is surely more diagnostic of what a koala is than for example *tree bear*. Finally, *informativeness* indicates whether both constituents are actually needed (and sufficient) to identify the meaning of the combined concept. In the case of *water lake*, adding the modifier *water* is at best unnecessary, if not confusing in most contexts.

In the Constraint Model, the interpretation of a noun compound is then assumed to be the most acceptable one, while acceptability is a function of these three constraints. Note that acceptability here refers to the acceptability of different *interpretations* of a given compound, not to the acceptability of the compound itself. However, it seems reasonable to assume that the plausibility (in terms of *meaningfulness*, as discussed previously) of a compound is a function of the acceptability of its interpretation: A compound for which a good interpretation can be obtained should be considered more plausible than one for which even the best interpretation is not very acceptable.

#### Distributional Semantic Models

In the theories of conceptual combination discussed so far, some major theoretical concepts remain underspecified. There remain free parameters, such as the dimensions and features a concept includes, and how exactly those are changed in a specific combination of a modifier and a head noun. Although models of conceptual combination have been successfully implemented computationally [11], [52], [9], these implementations rely on hand-crafted encoding of those parameters [56].

Distributional Semantic Models (DSMs) provide a possibility to address these issues. In DSMs, the meaning of a word is represented by a high-dimensional numerical vector that is derived automatically from large corpora of natural language ([57], [58], [59], for overviews on DSMs). For the remainder of this article, we assume that word meanings correspond to concepts ([60] provides a detailed discussion on this issue).

The core idea of distributional semantics is the *distributional hypothesis*, stating that words with similar meanings tend to occur in similar contexts [61]. This should also be reflected in the opposite direction: Words that appear in similar contexts should in general have more similar meanings than words appearing in different contexts. For example, the meanings of *moon* and *sun* can be considered to be similar as they often occur in the context of *sky*, *sun*, *universe*, *light* and *shine*.

By explicitly defining the notion of context, the distributional hypothesis can be quantified. The two most common approaches are to define context as the documents a word occurs in [62], [57], or as the words within a given window around the target term [63] (see [58], for the differences between these approaches).

We will illustrate the second option with a toy example. Assume we want to extract vector representations for the word *moon*. As relevant context words we take *sky*, *night* and *shine*, and we assume that two words are co-occurring if and only if they appear in adjacent positions in a sentence (technically, within an *1-word window*). Scanning through the corpus, we then find 2 co-occurrences of *moon* and *sky*, 5 co-occurrences of *moon* and *night*, and 3 co-occurrences of *moon* and *shine*. Therefore, we can derive the following vector representation for *moon*:

moon = (2, 5, 3)

The same procedure can be applied to other words as well. For example, counting co-occurrences between *sun* and *sky*, *night*, and *shine* might result in the vector

sun = (3, 1, 5)

If the same context words (in the same order) and the same corpus were used to construct two word vectors, these will live in the same *semantic space*. In this case, it is possible to approximate how similar two word meanings are, usually by computing the cosine similarity between the two respective word vectors, which is defined as

$$cos\left(a,b\right)=\frac{\sum_{i=1}^{n}a_{i}\cdot b_{i}}{\sum_{i=1}^{n}a_{i}\cdot \sum_{i=1}^{n}b_{i}}$$ (1)

for two *n*-dimensional vectors *a* and *b*. If there are only positive values in the vectors, as is the case for raw co-occurrence counts, the cosine similarity ranges between 0 (for orthogonal, that is unrelated vectors) and 1 (for identical vectors). In the example above, the cosine similarity between *moon* and *sun* is .71.

The vectors derived this way are typically further processed, by applying weighting schemes on the raw counts, as well as dimensionality reduction techniques [64], [65], [59]. The purpose of applying weighting schemes is to adjust for frequency effects: Usually, very frequent words (such as *and* or *was*) are less informative for the meaning of their surrounding words than infrequent words (such as *cardiology* or *xylophone*); furthermore, the similarity of two word vectors based on raw co-occurrence counts is considerably influenced by the words’ frequencies. The purpose of dimensionality reduction techniques, such as Singular Value Decomposition (SVD) or Non-negative Matrix Factorization(NMF), is to get rid of noise in the data, and to generate latent, underlying dimensions of meaning as context dimensions [57].

#### Distributional Semantics in Cognitive Science

Originally, DSMs were designed as a method in computational linguistics and natural language processing, but soon became popular in cognitive science, mainly due to the success of popular models such as Latent Semantic Analysis (LSA; [62], [57]) or the Hyperspace Analogue to Language (HAL; [63]).

It has been shown in numerous studies that DSMs are a psychologically plausible approach to meaning [57], [66], [67], [68], [69], [70]. Apart from being able to account for various empirical behavioural phenomena, such as predicting human similarity ratings [57] or priming effects [67], [71], there are also more theoretical ways in which DSMs can be aligned with psychological theories: They can encode properties of concepts [69], [72], and provide an account of how we learn, structure and abstract from our experience and induce relations that were not explicitly stated or observed [57].

It is hereby more a contingent property rather than a defining feature of DSMs that they seem to be centred around word co-occurrences. This is mainly due to the availability of large text collections and the tools to process them, which are mostly practical issues. In fact, DSMs can also be designed to encode extra-linguistic information, which has already been done successfully with visual information [73], [74]. Therefore, DSMs should be seen as a formal description of how experiential input is organized and information is structured in our minds, by considering the contexts in which a stimulus (in this case, a word) was or was not present, and the contextual similarity to other stimuli. Indeed, even when considering purely textual input, the view that DSMs can only capture textual similarity is somewhat misguided: Studies by Louwerse [75], [76] show that DSMs do not only encode linguistic information, but also world knowledge and even information that is usually considered to be embodied, such as spatial-numerical associations [77]. As an example for the encoding of world knowledge, [75] show that lexical similarities between city names in LSA correspond to the actual geographical distances between those cities. The observation that language encodes a lot of information about the actual world is highly plausible given that, in many cases, language is used to talk about the world.

Furthermore, an important point concerning the two possible representations of word meanings (or concepts) as high-dimensional numerical vectors (as in DSMs) and as lists of features (as assumed in models of conceptual combination) has been made in [66] (compare [57], for an earlier version of this idea). They show that there is actually a correspondence between those two representations, as a vector representation can be seen as a probability distribution over different semantic topics (see also [69]). Therefore, the dimensions which constitute the vectors in DSMs can be interpreted as semantic dimensions of the respective words, or concepts [57], although it might be difficult to name those dimensions on an individual basis. In conclusion, vector representations of meanings DSMs are *not* just to be seen as refined co-occurrence counts, and DSMs should not be taken as inventories purely encoding lexical statistics.

#### Composition in Distributional Semantics

At this point, we only discussed how meanings of single words are represented in DSMs. However, meanings can clearly also be assigned to more complex expressions, and models of meaning should account for that. Especially, it is important to be able to obtain meanings also for novel expressions that were not encountered before, since the possibility to generate novel combinations is an essential property of language.

Recently, the topic of *compositionality* in DSMs has received considerable attention [78], [79], [80], [81], [82]. The basic feature of compositional DSMs is that the vector representation of a noun compound lives in the same semantic space as the vector representations for single words, and it can be computed arithmetically on the basis of the elements in the expression (see the Methods section for technical details). In the case of noun compounds, the compound vector is therefore based on the modifier noun and the head noun. Importantly, such vectors can also be computed for compounds that were never attested in a corpus.

In general, the relation between the compound meaning and its constituents can be stated as

c=f(m,h) (2)

with *c* being the vector representation of the compound, *m* and *h* being some representation of the modifier and the head (not necessarily in vector terms, see Methods), and *f* being a function linking those representations. Note that this formulation is identical to other linguistic theories of compound meanings, for which a main objective is to identify the function *f* for a given compound [40].

This relation implies that each dimensional value *p*_*i*_ of vector *p* is itself dependent on the modifier and the head noun of the compound. Therefore, compositional models in DSMs are comparable to psychological theories of conceptual combination, which also assume that the dimensional values of the combined concepts are a function of the compound’s head and modifier (as described earlier).

In this perspective, we can see compositional methods for DSMs as an algorithmical formalization of conceptual combination: Instead of hand-crafted feature lists, concepts are represented as data-driven, high-dimensional numerical vectors; and the process of combination itself is formalized by applying arithmetical operations, resulting in a vector representation for the compound.

In summary, we assume that the product of the comprehension stage for a compound is as a vector, derived compositionally on the basis of the compound constituents. Following [66], this vector representation corresponds to sets of features of the combined concept.

### The Assessment of Noun Compound Plausibility

In a very recent study on the plausibility of novel adjective-noun phrases [31], it was found that human plausibility judgements could best be predicted by the similarity between the phrase meaning and the meaning of the head noun. These meanings were computed using compositional DSMs, as presented above, and the similarity was defined as the cosine similarity between the phrase vector and the head noun vector. This result goes in line with the view of conceptual coherence in terms of category memberships: If a combined concept, such as *sweet cake*, is similar to the head category (*cake*), it fits prior knowledge about that category, which makes it a plausible combination. On the other hand, the combined concept *muscular cake* is too dissimilar to the usual experience with cakes, and will therefore be considered more implausible. Note that, contrary to the other variables discussed so far in this section, this similarity between phrase and head noun actually needs a representation of the phrase meaning.

#### Plausibility Measures in Distributional Semantics

In the study in [31], several measures in distributional semantics for phrase plausibility were employed (also called *semantic transparency measures*). It has already been shown in other studies that such measures are useful in predicting the plausibility of adjective-noun phrases [83], [31] as well as word-affix combinations (such as *re-browse* vs *re-wonder*) [7], and resolving syntactic ambiguities for three-word compounds [84]. In this section, we will describe those measures and the rationale behind them

- **Head Proximity.** *Head Proximity* is defined as the cosine similarity between the expression in question and its head (in our case, between the noun compound and its head noun), so
  head_proximity=cos(c,h) (3)
  with *c* being the phrase in question, and *h* being the vector of the head noun. Hence, the head proximity indicates how related a compound meaning is to the meaning of its head noun, or how much this head noun meaning contributes to the compound meaning. In that, Head Proximity is related to the concept of analysability in linguistic theories of compounding [85], [86], which is defined as “the extent to which speakers are cognizant (at some level of processing) of the contribution that individual component structures make to the composite whole” [86] (p. 457). It has been argued that analysability is a gradual phenomenon and therefore a continuum rather than a binary notion; this is in line with our approach, which defines Head Proximity as a gradual cosine similarity.
  The general idea here is that a higher head proximity indicates a more plausible phrase. For example, if a *house boat* is still highly related to the concept of *boat*, one would expect *house boat* to be a rather plausible phrase.
  As discussed earlier, this assumption is in line with conceptual coherence, as an indicator of how well a combined concept can be fitted to prior experience with the respective head concept. Following the constraint of diagnosticity [9], combined concepts should be some instance of the category described by the head noun, or at least share a sufficient amount of features with it. Otherwise, the usage of another head noun to create the compound would have been a better choice.
- **Modifier Proximity.** The same notion of proximity between a phrase and constituent can also be applied to the modifier:
  modifier_proximity=cos(p,m) (4)
  with *p* being the phrase in question, and *m* being the vector of the modifier noun. The rationale of diagnosticity, as already discussed for Head Proximity, can be applied here: In order for a phrase like *house boat* to be plausible, it should also be related to the concept *house*, because there should be a reason that exactly this modifier is included in the phrase. *Therefore, the concept should be analysable with respect to the modifier, that is the modifier’s contribution to the compound meaning should be identifiable.*
  So far, we have argued that, according to the diagnosticity constraint, higher proximities between the constituents and the phrase should result in more plausible phrases. However, according to [9], the influence of diagnosticity is modulated by informativeness, that is whether both constituents are necessary and sufficient to constitute the intended compound meaning. Therefore, the relation between the proximities and plausibility might not be a linear one, or maybe not even monotonously positive. For example, it can be argued that in the case of rather non-informative compounds such as *water lake*, too close a relatedness between the constituent meanings and the compound meaning leads to relatively lower plausibility judgements.
- **Constituent Similarity.** *Constituent Similarity* is defined as the similarity between its modifier noun and its head noun:
  constituent_similarity=cos(m,h) (5)
  with *m* being the vector for the modifier, and *h* being the vector of the head noun. [56] found the LSA cosine similarity between the two constituents of a phrase to be predictive for its plausibility: This similarity was larger for typical adjective-noun pairs (such as *sharp saw*) than for atypical adjective-noun pairs (such as *mortal god*), and this similarity again was larger than for noun compounds. These differences correspond to differences in the ease of comprehension for these compound types, as indicated by human ratings, lexical decision reaction times, and classifications whether a compound is plausible or not [47].
  However, note that Constituent Similarity captures conceptual coherence only on the level of single word meanings: If the two concepts that are combined are coherent, the compound should be perceived to be more plausible as when they are incoherent. However, if the plausibility of a compound was only determined by the similarity between its constituents, it would be possible to judge it without having a representation for the compound meaning. This is hard to bring in line with the literature on conceptual combination.
- **Neighbourhood Density.** For each vector living in the semantic space, its *m* nearest neighbours are defined as those words having the highest cosine similarity with the said vector. *Neighbourhood Density* refers to the average similarity between a vector and these neighbours
  $$neighbourhood\_density=\frac{1}{k}\cdot \sum_{i=1}^{k}\cos(c,n_{i})$$ (6)
  with *c* being the (compound) vector in question, *k* being a fixed number of nearest neighbours to be considered, and *n*_*i*_ being the *i*th nearest neighbour to *p*.
  The idea behind selecting neighbourhood density as a measure for plausibility is the assumption that plausible expressions should live in a higher-density neighbourhood than implausible ones. The meaning of a more plausible expression should be quite similar to other, already known concepts, and it should be quite clear from that neighbourhood which meaning the expression conveys. A less plausible expression, on the other hand, should be fairly isolated from other concepts, which makes it hard to tell what it means.
  Since neighbourhood density is a measure of how similar a concept is to various already known concepts, it is in line with the notion of conceptual coherence as a determinant of plausibility.
- **Entropy.** Entropy is a prominent concept in information theory, indicating how far a (probability) distribution deviates from a uniform distribution. For an *n*-dimensional vector *p* with a value of *p*_*i*_ on the *i*th dimension, it is defined as
  $$entropy=log\left(n\right)-\frac{1}{n}\cdot \sum_{i=1}^{n}p_{i}\cdot log\left(p_{i}\right)$$ (7)
  High values of entropy indicate a distribution that is close to a uniform distribution, while lower values indicate a more diverse distribution, with peaks in some dimensions and very low values in others.
  Entropy can be hypothesized to predict the plausibility of an expression from its vector: A vector for a plausible expression should have high values on the dimensions that are highly diagnostic for the concept, and low values on other, irrelevant dimensions. Following [66], such a vector represents a concept that has defined features. On the other hand, a vector that is very close to a uniform distribution has no specific dimensions with which the respective concept is likely to occur. Therefore, such a concept has no distinct features, and should therefore be implausible.

#### Outlines for the Present Study

In this study, we want to investigate which factors determine the plausibility of noun compounds. To achieve this, we employ compositional methods in distributional semantics in order to obtain formalized vector representations for these compounds, and use different plausibility measures that capture different aspects of conceptual coherence in compounds.

In this, our study has a similar approach as the study in [31]. However, we extend this study in several respects: First, we focus on noun compounds instead of adjective-noun phrases and therefore to another class of expressions and conceptual combinations. While most literature on conceptual combination accounts for both cases [16], some models, such as the Selective Modification Model [44], [11] cannot account for noun compounds, as discussed earlier.

Secondly, while [31] have concentrated on plausibility judgements only for unattested and hence novel adjective-noun phrases (such as *spectacular sauce*), we want to investigate attested as well as novel noun compounds. This will provide us with a more comprehensive and general picture of what influences plausibility judgements, for a variety of differently familiar compounds.

Finally, the focus of the study in [31] was to find out which compositional method in combination with which plausibility measure predicted human plausibility ratings best. This approach gives computationally efficient results, but does not take into account whether different measures play differently prominent roles in judging plausibility. Furthermore, potential interactions between the measures are neglected. Such interactions are suggested in [9], by assuming that diagnosticity and informativeness should modulate each other. In our study, instead of choosing the single best predictor, our aim is to model plausibility judgements for noun compounds with the best-fitting combination of plausibility measures, including possible non-linear effects and interactions.

## Method

### Data set

We employed the data set provided in [30] for our analysis. This data set contains plausibility ratings for 2,160 noun compounds.

These noun pairs were generated by first taking the 500 most concrete nouns provided from various imageability studies. Of all the possible pairwise combinations of those 500 nouns, those were retained that (a) appeared at least once in the 7-billion-word USENET corpus [87] and (b) were considered not problematic by the authors (for example, apparently nonsensical compounds were removed). This procedure resulted in 1,080 attested noun pairs.

The second half of the item set was obtained by reversing the word order of those 1,080 noun pairs. For example, since the pair *bike pants* is included as an attested compound, its counterpart *pants bike* also is included in the final item set. As a result of the selection process, these reversed items did either not appear in the USENET corpus, or were considered to be problematic.

This structure of the data set is especially interesting for two reasons: Firstly, the reversed-order compounds are not attested in a large corpus, which indicates it is unlikely that the participants in the study in [30] have ever encountered one of them before. Therefore, they could not rely on a stored entry in their lexicon to identify the meaning of those compounds, and had to interpret them in a compositional fashion. Secondly, given the asymmetry of compounds, compounds with reversed-ordered constituents are not derivationally related, and the two orders result in often very different interpretations, if they are interpretable at all [22], [23]. Thus, in order to come up with a plausibility rating for these compounds, the meaning for the reversed-order compounds had to be interpreted on-line, by relying on a compositional process, and is not the same as for their attested counterparts.

For the resulting set of 2,160 noun pairs, plausibility ratings were obtained through an online questionnaire. Participants were asked to indicate how meaningful the pair was *as a single concept*, ranging from 0 (makes no sense) up to 4 (makes complete sense). The mean rating for each noun pair was then obtained by averaging over those plausibility ratings after the removal of outliers (see [30] for further details).

### Word Vectors—The Semantic Space

In order to obtain vector representations for the compounds on which plausibility measures can be applied, we first have to set up a semantic space from a source corpus. This semantic space is a matrix containing all the word vectors needed for the analysis as row vectors, and a fixed number of semantic dimensions as column vectors (as described in the *Distributional Semantic Models* section). The following section will describe the construction of the semantic space employed in this study in further detail.

#### Corpus

The corpus used to derive the semantic space resulted from the concatenation of three corpora: The British National Corpus (http://www.natcorp.ox.ac.uk/), the ukWaC corpus obtained from web sources (http://wacky.sslmit.unibo.it/) and a 2009 English Wikipedia dump (http://en.wikipedia.org). This corpus contains a total of about 2.8 billion tokens—an amount that is comparable to a lifetime’s total language experience (which is which is estimated to be about 2.2 billion words; [88], [89]). The corpus has been tokenized, lemmatized, and part-of-speech tagged using TreeTagger [90] and dependency-parsed using MaltParser (http://www.maltparser.org).

We only considered the lemmatized version of each token in our analysis (i.e., different word forms of *monkey*, such as *monkey* and *monkeys*, will both be mapped onto the lemma *monkey*). For a discussion on lemmatization, see [59]. In the remainder of this section section, we refer to those lemmata when we speak of *words*.

#### Vocabulary

In a semantic space, each row gives the vector representation for a word. Word vectors were computed for the following words:

- The 20,000 most frequent content words (nouns, verbs, adjectives, adverbs) in our source corpus.
- The constituents of the word pairs in the data set from [30]
- All the words that were part of any training set for the composition methods we employed (see the section on *Composition Methods and* S1 Appendix for details).

In total, this resulted in 27,090 words populating the semantic space (i.e. 27,090 row vectors).

#### Constructing the Semantic Space

The context dimensions (i.e., the columns of the semantic space) were set to be the 20,000 most frequent content lemmata (nouns, verbs, adjectives, adverbs) in the source corpus. Therefore, the initial semantic space is a 27,090 × 20,000 matrix.

The cells of this semantic space were filled up by sliding a ±2-word context window over the corpus [63]. Each word in the vocabulary was therefore considered to co-occur with the two context words preceding and following it. For each co-occurrence of vocabulary word *i* with context word *j*, the value in cell (*i*, *j*) of the semantic space was increased by 1. Only co-occurrences within sentences were counted. The procedure results in a *raw count matrix*.

In a next step, a positive *Pointwise Mutual Information* (PMI) weighting [91] was applied to this raw count matrix. The PMI measure is a widely used word association measure and defined as follows:

$$PMI\left(a,b\right)=log(\frac{p(a,b)}{p(a)*p(b)}),$$ (8)

with *a* and *b* being two words, *p*(*a*, *b*) being their probability of co-occurrence, and *p*(*a*) and *p*(*b*) being their marginal probability of occurrence. PMI therefore measures whether the actual co-occurrence probability of two words is higher than their probability of randomly co-occurring. *Positive* PMI (PPMI) is a variation of this measure where resulting negative PMI values are set to zero. It has been shown that applying PPMI weightings to the raw counts considerably improved the performance of DSMs [64].

In a last step, *Non-Negative Matrix Factorization* (NMF) [92] was used to reduce the dimensionality of the weighted count matrix. Dimensionality reduction techniques, especially Singular Value Decomposition (SVD), are used very often in DSMs, and improve their performance considerably [57], [93], [65]. We decided to use NMF instead of SVD, as it was shown to give better empirical results [92]. Furthermore, it has been shown that employing Non-negative Matrix Factorization (NMF) as a dimensionality reduction technique on window-based semantic spaces produces dimensions that can also be interpreted in a probabilistic fashion as a distribution over different topics or features [94], as is the case for topic models [66]. We also performed the computations reported here using SVD, which gave very similar results. NMF is similar to SVD, with the difference that all resulting vectors only contain non-negative values (which is not necessarily true for SVD). The algorithm was set to reduce the weighted count matrix to a semantic space with 300 dimensions, based on previous findings [57].

The free software toolkit DISSECT [95] was used to perform the computations needed to construct the semantic space.

### Obtaining Compound Vectors

In order to obtain vector representations for the compounds in the data set, we employed various composition methods [79], [80], [81]. In a pre-test (see S1 Appendix), the best results were obtained when the modifier noun was applied as a *lexical function* to the head noun [81], [82]. In this paragraph, we will describe this method in further detail.

In this approach, composition is seen as applying a linear function to a vector, so that

c=M·h (9)

with *c* being the *n*-dimensional compound vector, *h* being the *n*-dimensional vector representation of the head noun, and *M* being an *n* × (*n* + 1)-dimensional matrix (an *n* × *n* transformation matrix with an *n* × 1 intercept) specifying how the modifier changes the meaning (i.e., the vector) of the head.

The vectors for the head noun are taken from the semantic space. The matrices for the modifiers are then computed by employing a regression-based approach, using training sets. Therefore, how a modifier noun changes the meaning of head noun when applied to them is learned from instances where that noun is used as a modifier. We will illustrate this using an example:

Assume one wants to derive the matrix representation for the modifier noun *moon*. In this case, one selects from the corpus different noun compounds containing that modifier, for example *moon calendar*, *moon landing* and *moon walk*. For those compounds, it is possible to compute *oberserved* phrase vectors, by treating them like a single word and counting their co-occurrences with the context dimensions.

At this point, we have vector representations *v* for the head nouns (*calendar*, *landing*, and *walk*), as well as vector representations *p* for the noun compounds (*moon calendar*, *moon landing* and *moon walk*). The cell values of the matrix *U* can now be estimated solving a regression problem. A matrix for a modifier is thereby estimated by minimizing the the Euclidean norm between the observed vectors for the compounds in the training set and their composed vectors as computed by Eq 9.

The matrices obtained this way indicate how much each dimension of the head noun, when combined with the modifier, influences each dimension of the compound. Once a matrix is obtained, it can be applied also to vectors for head nouns that were not part of the training set, and hence be used to obtain vector representations also for non-attested noun compounds. This composition method has already been successfully applied in psycholinguistic studies [7], [31]

#### Training the Lexical Functions

The training set for the Modifier Lexical Function consisted of all the noun pairs in the corpus (a) where the *first* noun appeared as a constituent in the item set (and hence as a modifier, in the attested or the reversed order), and (b) that occurred at least 20 times in the corpus. There are 391 different modifiers in the item set. Since estimations are unreliable if there are not enough training items for a specific modifier, we removed 163 modifiers for which there are less than 50 different training pairs in our source corpus. For the remaining 228 modifiers, a total of 52,351 training pairs were found, with up to 1,651 different training pairs per modifier noun. Pairs that were part of the data set were not used as training items.

The lexical function matrices were estimated and compound vectors were computed using DISSECT [95].

Since we eliminated 163 modifiers from the data set, we obtained 1,699 compound vectors (881 for attested and 818 for unattested compounds).

### Predicting Variables

#### Plausibility Measures

As variables for predicting the plausibility of the compounds, we employed Neighbourhood Density (setting the size of the neighbourhood to *k* = 20 without tuning) and Entropy, computed on the 1,699 compound vectors that we derived compositionally. Head Proximity and Modifier Proximity were also computed on these compound vectors, with the vector representations for the head noun (or modifier noun, respectively) obtained from our semantic space. Furthermore, we computed the Constituent Similarity between modifier noun and head noun from their vector representations in our semantic space.

#### Covariates

In addition to the plausibility measures, we considered several linguistic covariates:

- *Length* (in letters) for modifier and head nouns
- *Logarithmic frequency* of modifiers, heads, as well as the modifier-head pairs in *both* orders according to the 201-million-word SUBTLEX corpus [96]. We avoid the term *compound frequency* and use *modifier-head pair frequency* in this article, since every occurrence of modifier and head next to each other, not necessarily as a compound, is counted for this frequency. Thus, for the compound *tree apple*, we considered the logarithmic frequency of both *tree apple* as well as *apple tree* as a covariate. To deal with zero frequency words and bigrams, we used the Laplace transformation for frequencies [97].
- *Family size* for modifiers and heads, according to our source corpus. Family size specifies in how many different compounds a modifier noun is used as modifier, or a head noun is used as head
- *Pointwise Mutual Information* between the modifier noun and the head noun [91]. This variable specifies how the probability of two nouns actually occurring together relates to the probability that they randomly occur together, and is a measure for the association between two words.

## Results

Since the constraint of informativeness suggests possible non-linear effects of some plausibility measures, we employed Generalized Additive Models [98], [99] to analyse the plausibility data, using the package *mgcv* [100] for R [101].

### Baseline Model

After a first inspection, we deleted family sizes from our set of covariates, since they were highly correlated with the respective word frequencies (*r* = .68, *p* < .001 for modifier nouns, *r* = .64, *p* < .001 for head nouns).

We then identified a baseline model containing fixed linear effects for the covariates, as well as random effects for head nouns and modifier nouns. To achieve this, we started from a model containing all those effects (see *Covariates* in the Methods section). Only linear effects for the covariates were considered in order to keep the baseline model simple. We then checked which of the parameters in this model contributes significantly to predicting the data, by performing Wald tests for each linear fixed effect in the model. Non-significant parameters were removed from the model. By counter-checking with additional Likelihood-ratio tests, we ensured that this baseline model could not be significantly improved by adding further fixed linear effects for any covariate (this is also true for the initially excluded family sizes), and that removing any of the included effects significantly worsens the model. Table 1 shows which covariate parameters remained in the baseline model, and gives their parameter values in the *final* model.

**Table 1:** Parameter values for parameters added to the model. *te()* indicates non-linear (tensor) interactions.

*Linear Coefficients*				
Coefficient	Estimate	SE	*t* value	*p*
Intercept	1.580	0.126	12.514	< .001
Modifier Length	0.100	0.023	4.284	< .001
Reversed-ordered Pair Frequency	-0.106	0.149	-7.075	< .001
PMI	0.167	0.043	3.840	< .001
*Non-Linear Coefficients*				
Coefficient	Estimated *df*	Residual *df*	*F* value	*p*
Head Proximity x Modifier Proximity	16.442	18.256	9.544	< .001
Modifier Proximity x Constituent Similarity	1.689	8.000	2.845	< .001
Constituent Similarity x Pair Frequency	6.439	7.843	46.074	< .001

### Testing for Effects of the Plausibility Measures

Starting from the baseline model, we tested for effects of the plausibility measures in a step-wise procedure. In each step of this procedure, we estimated a set of different models, each containing all the parameters of the model from the previous step, plus an additional effect for a plausibility measure that was not already part of the model. Then, Likelihood-ratio tests were used to test whether any of those models predicted the data significantly better than the model from the previous step. If this was the case, we continued with the next step, where this procedure was re-applied. If at any given step multiple models predicted the data significantly better, we opted for the model with the lowest Akaike Information Criterion (AIC) [102]. Interaction effects were tested for if the respective lower-order effects were already part of the model. After adding the effects for the plausibility measures to the model, we further tested whether any of those effects was influenced by the familiarity with the compounds (as approximated by the frequency of the modifier-head pair).

Further details on this step-wise procedure, as well as the order in which parameters were added to the model, can be found in S2 Appendix.

**Fig 1:**
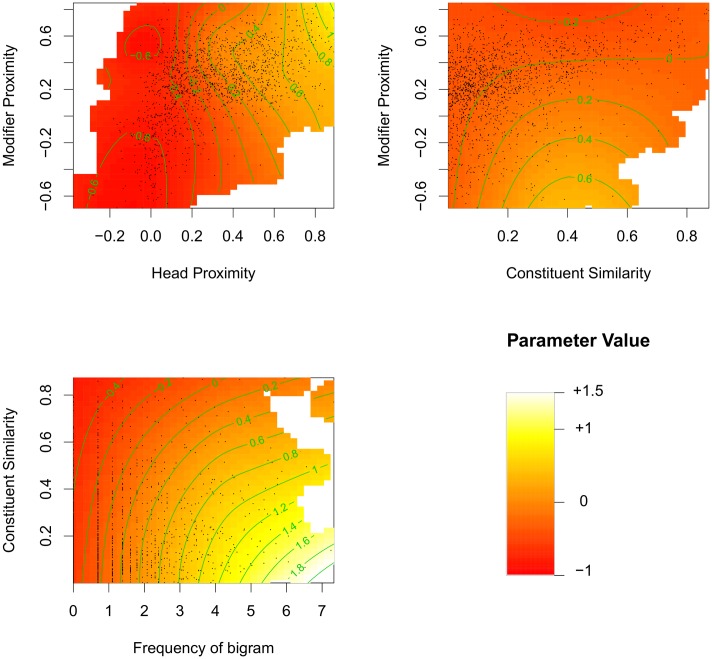
Heat maps for the non-linear interaction effects including plausibility measures. The colours indicate parameter values (i.e., predicted deviation from the mean), the points show the data points from which the model was estimated. *Upper left:* Interaction between Head and Modifier Proximity. *Upper right:* Interaction between Modifier Proximity and Constituent Similarity. *Lower left:* Interaction between frequency of bigrams and Constituent Similarity. *Lower right:* Legend.

The parameter values for the final model resulting from this procedure are given in Table 1. This model contains three non-linear interaction effects, between Head Proximity and Modifier Proximity, between Constituent Similarity and Modifier Proximity, as well as between Constituent Similarity and the frequency of the modifier-head pair. Heat maps for these effects are displayed in Fig 1.

### Model criticism

After establishing a final model for the data in a step-wise procedure, we tested whether this model is heavily influenced by outliers, whether the complex non-linear effects are indeed necessary in the model, and whether the effects are caused by some values with negative Modifier Proximities or Head Proximities.

To test for the first possibility, we removed from our data set all data points which deviated more than 2.5 standard deviations from the model predictions (these values can be considered outliers), and then fitted our final model to this new data set. As indicated by Wald tests performed for the parameters this model, all included parameter terms are still significant. Furthermore, the explained variance is even higher in this case (*R*^2^ = .67 for the model estimated on the whole data set vs. *R*^2^ = .71 for the model estimated on the data set where outliers were removed). This supports the view that our final model does not contain effects caused by some outliers.

Additionally, Likelihood-Ratio tests show that the model predictions are significantly worse if any non-linear interaction term is replaced by a linear interaction of the same two variables. Therefore, the non-linearity of those effects is necessary in the final model. We also re-estimated the final model on a data set where data points with negative Modifier Proximity and Head Proximity values were removed (since it is not clear how to interpret negative cosine similarities). Again, all parameters in the final model are significant (as indicated by Wald tests), and the non-linear effects could still not be replaced by linear interactions (as indicated by Likelihood-ratio tests).

## Discussion

We derived vectors representing the meaning of attested and reversed-order compounds, using compositional methods in distributional semantics, in order to predict human plausibility ratings for these compounds. From those vectors we derived several plausibility measures. We found that three non-linear interactions involving those measures contribute to predict the plausibility ratings: An interaction between Head Proximity and Modifier Proximity, a negative interaction between Constituent Similarity and Modifier Proximity, and a negative interaction between Constituent Similarity and the frequency of the modifier-head pair (i.e., the familiarity with the compound). In the following sections, we will discuss these interactions.

Note that what follows are descriptions of the results we found, expressed and interpreted in psychological terms. We then propose a way to integrate these findings into a processing account of plausibility judgements. Hence, empirical hypotheses can be derived from our results; it remains subject to further, experimental studies, to determine if the processes we describe actually play a role in the psychological assessment of noun compound plausibilities.

### Interactions of Plausibility Measures

#### Head Proximity and Modifier Proximity

As can be seen in the upper left panel of Fig 1, Head Proximity has a positive effect on the plausibility of compounds: The higher the Head Proximity is, the higher plausibility ratings tend to be. Since this statement holds for all levels of Modifier Proximity, this is a general positive effect of Head Proximity.

Considering that the role of the head noun in a compound is to define the semantic category the compound belongs to [19], this effect can be explained as an effect of the ease of categorization. In general, compounds are rated as more plausible the closer the respective combined concept is to the category (or concept) denoted by the head noun, that is the easier it is to interpret them as an instance of this category. This is in line with the common finding that the relatedness to a category prototype is a major determinant of whether a specific concept is a member of that category [103]. As discussed previously, distributional semantics leads to representations of concepts that can be interpreted as prototype schemata. Note that, in such an interpretation of our results, the view that the compound is a hyponym of the head and therefore a member of the head category is very prominent. This is not strictly speaking logically true for all compounds, since there exist exocentric compounds such as *metalhead* (but see [27], [104] for critical views on the topic of exocentricity). However, this does not imply that our analysis is restricted to endocentric compounds only. Instead, we assumed as a working hypothesis in the present study that human judge apply the same mechanisms for judging the plausibility of noun compounds of different categories. The empirical validity of this working hypothesis has to be sorted out in future research.

Examples for compounds with low and high Head Proximity values can be seen in Table 2. As can be seen from these examples, it is much easier to identify the compounds with high Head Proximities as members of the head noun category, while the same is very hard (or almost impossible) for compounds with low Head Proximities.

**Table 2:** Example items for compounds with low vs. high Head Proximity Values.

Low Head Proximity (< .1)	High Head Proximity (> .6)
diamond tennis, milk mouse, guy bird, pie moon, pen bull, pool sun,	orange juice, golf shirt, rose garden, hotel cafe, beach sand, island prison, bell tower

However, this effect of Head Proximity is strongly modulated by the Modifier Proximity. This interaction emerges in two patterns (see the upper left Fig 1). First, the effect of Head Proximity is steeper if the Modifier Proximity is medium-high, so already small raises of Head Proximity come with higher plausibility ratings. Stated in other terms, plausibility ratings drop off if the Modifier Proximity gets too high or too low, in comparison to medium-high Modifier Proximities (except for very high Head Proximities). The notion of informativeness [9] can be applied to explain this effect: If the meaning of a modifier is too distant from the compound meaning, it is hard to understand how exactly the modifier contributes to the compound. This difficulty comes with relatively low plausibility ratings. If, on the other hand, the modifier is too closely related to the compound, it can be considered as redundant, and there is no justification to include it in the compound at all. This redundancy violates the assumption that compounds should be informative, which comes with lower plausibility ratings.

That redundancy has negative effects on the interpretability of noun compounds has already been noted in [5], who specifies three conditions that cause redundancy: The modifier and the head noun refer to the same set of entities (e.g., *lad boy*); the set of entities referred to by one constituent is a proper subset of the set referred to by the other constituent (e.g., *horse animal*); or every instance of the head category is necessarily or typically an instance of the category denoted by the compound (e.g., *water lake*).

Note that, in our study, the representations for the compounds were derived compositionally from their constituents. In that light, Head Proximity and Modifier Proximity can be seen as a proxy of the contribution of the head noun and modifier noun to the combined concept: A high Head Proximity indicates that the meaning of head noun contributes highly to the compound meaning, as does a high Modifier Proximity with respect to the modifier (those two are not mutually exclusive, it can be the case that both constituents contribute highly or almost nothing to the combined concept). Therefore, our results indicate that redundancies occur when the contribution of the *modifier noun*, but not the head noun is too high *in the combination procedure*.

This point can be illustrated with some example items, see Table 3. As can be seen, items with an “optimal” medium Modifier Proximity appear to be intuitively plausible. On the other hand, for items with a low Modifier Proximity, the contribution of the modifier to the compound is not clear at all; and items with a high Modifier Proximity appear to be highly redundant.

**Table 3:** Example items for compounds with different Modifier Proximity values, all with medium-high Head Proximity values (between .3 and .5).

Low Mod. Proximity (< .2)	Medium Mod. Proximity (.4 − .6)	High Mod. Proximity (> .6)
road bed house rainbow school dog boot screen book mirror	soup chicken school book bike seat beach house ship engine	sun summer engine vehicle shirt dress engine car school university

However, for compounds with a high Head Proximity value, while the drop-off in plausibility for low Modifier Proximities is still present, the effect for high Modifier Proximities is different: For these items where both Head and Modifier Proximity are high, the model predicts very high plausibility ratings. This effect might truly be one of a specific interaction between Head Proximity and Modifier Proximity, in that high values on *both* do not invoke the informativeness issues discussed before. More specifically, once the Head Proximity reaches a certain threshold (of about.65 in our data), the drop-off for high Modifier Proximities no longer appears. In those cases the high Head Proximity could just override those issues, since the compound is very easy to interpret as an instance of the head category, which might be more important than having an informative phrase ([9] also postulate that informativeness plays a subordinate role compared to the constraints of plausibility and diagnosticity).

Upon inspecting these items, however, we find a relatively large amount of lexicalized compounds: *rain cloud*, *swimming pool*, *cheese cake*, *chicken salad* and *river valley* are amongst them. We therefore propose to be cautious with regards to the generic interpretation of this effect, since it might be driven by other factors such as lexicalization.

#### Constituent Similarity and Modifier Proximity

The upper right panel of Fig 1 shows the second interaction effect, between Constituent Similarity and Modifier Proximity. This effects consists of two main components: We find no effect for Constituent Similarity if the Modifier Proximity is above a certain threshold (about.4). Below that threshold, we find a positive effect for Constituent Similarity. For most items, this effect only predicts a very small gain in plausibility, although it is little bit higher if the Modifier Proximity is very low. Note that, although the model predicts drop-offs in plausibility for highly similar constituents, there are no data points after these drop-offs the model could be fitted on. Therefore, these drop-offs are most likely artefacts caused by the smoothing techniques used to estimate the model.

The small positive effect of Constituent Similarity is in line with the findings of [56] that more similar constituents predict more plausible compounds. However, as indicated by our analysis, this is not the case for all compounds, since this effect is absent if the Modifier Proximity exceeds a certain threshold (it should be noted here that [56] also conclude in there study that there is more to conceptual combination than just the similarity between constituents). We propose two explanations for this interaction:

The first possibility is that Constituent Similarity information is only used when the Modifier Proximity is low, that is when it is not clear how the modifier meaning contributes to the compound meaning. Such an interpretation assumes a positive effect of Constituent Similarity, but only for low Modifier Proximities. In that case, Constituent Similarity might help in overcoming interpretation difficulties that are caused by the opaqueness of the compound with regards to the modifier. If on the other hand the modifier’s contribution to the phrase meaning is sufficiently clear, there is no need to use this information, since the compound is already interpretable enough, and there is no need to consider Constituent Similarity.

Example items with low vs. high values on Modifier Proximity and Constituent Similarity that are in line with this interpretation can be found in the upper four cells of Table 4. For items with high Modifier Proximity values, such as *baby rabbit*, it is intuitively clear how the modifier contributes to the compound meaning, and therefore no further information on the similarity between modifier and head noun needs to be considered. On the other hand, for items with low Modifier Proximity values it might not be completely obvious how the modifier contributes to the compound meaning (is a *pie salmon* a rather round kind of salmon, or a salmon filled with something, or a salmon to be put in a pie?), but the general similarity between the constituents (both are some kind of food) makes it easier to align and combine them into a single concept.

**Table 4:** Example items for compounds with different low vs. high Modifier Proximity values, crossed with low vs. high Constituent Similarity values.

	Low Mod. Proximity (< .4)	High Mod. Proximity (> .4)
Low Const. Sim. (< .4)	building car, ship cow, meat cat, hill foot	phone car, salad island, sea lion, fox mask
High Const. Sim. (> .4)	pie salmon, dish oven cloud smoke, dog bull	nut milk, soup pot, baby rabbit, mountain lake
		meat pig, child infant, bed mattress, door kitchen

The second possibility to explain the interaction again considers the notion of informativeness, similar to our interpretation of the first interaction. Under this interpretation, we assume that Constituent Similarity generally has a positive effect on plausibility, but this effect is overshadowed by redundancies that occur when Modifier Proximity exceeds a certain threshold. In this case, the generally positive effect of Constituent Similarity and the negative effect caused by redundancies cancel each other out, and therefore we do not find a positive effect. Therefore, this second interpretation assumes a negative effect of high Modifier Proximity values that counteracts a positive effect of Constituent Similarity. Examples for this explanation can also be seen in Table 4, in the lower part of the bottom right cell, and include cases such as *child infant*. Of course, the similarity between *child* and *infant* is obvious, but the modifier *child* does not provide any semantic contribution to the compound, over and above the one brought upon by the head *infant*.

However, it is surely possible that both of the proposed mechanisms play a role in our study, and contribute to the pattern of results we found.

#### Constituent Similarity and Pair Frequency

The third interaction, between Constituent Similarity and the frequency of the modifier-head pair, is shown in the lower left panel of Fig 1. As can be seen there, the pair frequency has a positive effect on the compound plausibility; however, this effect becomes smaller the more related the constituents are to one another.

It is a common finding that frequency (i.e., familiarity) has a positive effect on plausibility of noun compounds [105], [30]. Our results extend these findings, as we find that this effect is modulated by the similarity between the head and the modifier (without considering Constituent Similarity, our model would also have identified a positive main effect for this frequency, see S2 Appendix).

We explain this effect analogously to the first explanation offered in the previous section: Information about frequency is used more as the compound becomes less coherent, in terms of the similarity of its constituents. This might indicate that humans draw back to the very basic property of familiarity if it difficult to see how the constituents of the compound relate to one another. However, note that the model does not actually predict *lower* plausibility ratings for highly frequent items with high Constituent Similarities, but only a smaller boost in plausibility as compared to items with low Constituent Similarities.

Similarly to the previous sections, we present some item examples for this effect in Table 5. The examples with high Constituent Similarities but low frequencies such as *door cabin* show that, while the constituents are clearly somehow related to one another, the fact that those compounds are virtually never used results in a “strangeness” makes it hard to judge them as being plausible.

**Table 5:** Example items for compounds with different low vs. high Constituent Similarity values, crossed with low vs. high frequency values.

	Low Const. Similarity (< .4)	High Const. Similarity (> .4)
Low Frequency (logFreq < 1)	guy field, engine cat, leg bike, lake deer face bed, pipe book	door cabin, nut banana, arm sword, dish cheese, soap television
High Frequency (logFreq > 1)	tea bag, police dog, rock star, sea lion star hotel, bell tower	chicken soup, sea water, summer holiday, baby boy, chocolate cake

Furthermore, considering the high-frequency items, it is clear on an intuitive level that items from both groups are frequently used. Note also that the first group contains some idiomatic compounds (such as *rock star* and *sea lion*) for which the relation between the constituents is not very clear without knowing what the compound describes. To interpret those compounds, readers might therefore heavily rely on the familiarity with the compound to judge its plausibility. For compounds such as *chocolate cake*, on the other hand, the relation between the constituents is quite obvious, and there is no need to rely on stored knowledge about the combined concept to interpret them.

Another possible explanation for the negative relation between Constituent Similarity and plausibility of the compounds could be the claim in [5] that too similar constituents could result in implausible compounds. However, [5] explicitly refers to highly similar, but mutually exclusive constituents, such as *butler maid* or *husband wife*. Upon inspecting the items with high Constituent Similarities, we did not find such items (except for—maybe—*tea coffee* and *coffee tea*, with a Constituent Similarity of .86). Therefore, this explanation does not hold for our results.

### Integrating the Results

In the original study presenting the data set we analysed, [30] also used a number of lexical variables (lengths, frequencies, association ratings and LSA cosine similarities for compound constituents) to predict the plausibility ratings for the compounds. They found significant effects for the compound length, the modifier-head pair frequency, the summed constituent frequencies, and LSA cosine similarities between the constituents. Our results largely resemble those obtained in [30]: Our baseline model includes a term for the modifier length (Graves et al. only examined the length of the whole compound, and not constituent lengths, therefore it is possible that their compound length effect is actually driven by modifier length), and modifier-head pair frequency is a powerful predictor also in our baseline model. In our step-wise modelling procedure, it turned out that this measure is part of an interaction with Constituent Similarity. This Constituent Similarity (in terms of LSA cosine similarities) also was found to be predictive for plausibility ratings in [30]; however, interactions were not considered in their model. Contrary to the original study, we did not find an effect of constituent frequencies, which might be caused by the facts that our baseline model includes additional variables that were not considered by Graves et al. (namely reverse-ordered pair frequency and PMI), that we used a corpus to obtain the frequencies (SUBTLEX, van Heuven et al., 2014, which is better suited for psycholinguistic purposes), or that we used a different modelling technique (Generalized Additive Models with random effects instead of standard multiple regression; random effects for constituents are a likely candidate to explain the absence of constituent frequency effects).

In their recent study on the plausibility of adjective-noun phrases, [31] employed some of the plausibility measures we used in this study. Similar to us, they also found that the best measure to predict plausibility was the relatedness between the phrase and the head noun of the phrase. However, these authors also found Neighbourhood Density to predict their data well, whereas this measure is not included in our final model. These differences are best explained by the differences in the data analysis: [31] employed a range of different models that each included only one plausibility measure, while in this study we estimated one model that includes different measures and their interactions. In fact, in the first step of our step-wise analysis, Neighbourhood Density was also found to significantly improve our baseline model, however not as much as Head Proximity (compare also S1 Appendix, which shows that the simple bivariate correlation between Neighbourhood Density and plausibility ratings is relatively high). Up to that point, our results are perfectly in line with the results obtained in [31]. However, at no point in the step-wise procedure we employed Neighbourhood Density did improve the model fit over and above the contribution of other plausibility measure, and hence it was eventually not included in the model. Therefore, while Neighbourhood Density as a single variable does predict plausibility ratings to some extent, it does not seem to explain anything over and above the other measures that are included in our model. Interestingly, [7] came to the same conclusion concerning Neighbourhood Density.

In their study, [7] investigated plausibility ratings for novel affixed words (such as *sketchable* and *unmessianic*), also using some of the measures employed in our study. They found Stem Proximity, that is the relatedness between the meaning of the affixed word (for example *sketchable*) and its stem (*sketch*), to predict these ratings. The meanings for the affixed words were also obtained using the Lexical Function method, with the affixes being Lexical Functions to be applied on the stem vectors. Interestingly, they found a quadratic (i.e., non-linear) effect of Stem Proximity, in that the highest ratings were found when Stem Proximity was medium-high. Thus, while this measure more resembles our Head Proximity (since both the head nouns and the stems are conceptualized as vectors on which a Lexical Function is applied), the pattern of results corresponds closely to our findings for Modifier Proximity.

As can be seen, our results differ to some extent from those obtained in [7]. It should however be considered that affixed words differ from noun compounds in many respects. The stems of those affixed words can belong to different syntactic categories (verbs, adjectives, and nouns), while noun compounds only consist of nouns. Additionally, affixation can change the syntactic category a word belongs to (*harass* is a verb, while *harassable* is an adjective), which is not the case for noun compounds, which are still nouns. Furthermore, a critical difference is that affixes themselves do not have a lexicalized meaning, as opposed to both constituents in noun compounds. This last point might explain the difference in the Proximity effects, in that Stem Proximity in affixed words captures aspects that overlap both Head Proximity and Modifier Proximity in noun compounds, since the stem is the only lexicalized part of an affixed word. Especially the redundancy effects we found for Modifier Proximity might be submerged into Stem Proximity. Due to these differences between affixed words and noun compounds, some factors that play a role for judging the plausibility of one kind of construct might not be relevant for the other.

Furthermore, [7] also found the plausibility ratings to be predicted by Vector Entropy, which we did not find in our study (at no point in our stepwise procedure did Entropy significantly improve the model, and the bivariate correlation between Entropy and plausibility ratings is not significant, see S1 Appendix).

However, there are also some parallels between our findings on noun compounds and the results on affixed words. Taken together with the fact that a very similar compositional method can be successfully applied for both cases, this could imply that some common underlying mechanisms guide the semantic composition and interpretation of these two classes of expressions. For a similar conclusion on the *processing* of complex words, see [106].

Taken together, the studies discussed here and our results in this study suggest that the strong predictors for plausibility, that emerge reliably in all the studies, are those that involve a comparison between the original constituent semantics (head noun, modifier nouns, stems) and the new, combined meaning of the complex expression (adjective-noun pair, noun compound, affixed word). However, our results show that this pattern is more complex than previous studies indicate, since there is a complex interplay involving those predictors.

We can build on the view that the relatedness between the complex expression and its constituents represent the contribution of the constituent meaning to the combined meaning to interpret these findings: In complex expressions, the constituents’ contribution to the complex expression is a critical factor for the perceived plausibility of the expression. This implies that, while judging plausibilities, humans represent both the constituent meanings and the combined meaning, and access how the former contribute to the latter. This apparently takes place for different types of complex expressions, and might therefore be an important underlying mechanism in judging plausibilities.

### General Discussion

#### A Mechanism for Plausibility Judgements

Taken together, our result suggest a mechanism such as follows that guides judgements on the plausibility of noun compounds: Initially, a representation for the meaning of the compound is constructed compositionally. As this representation is obtained, it is checked to what extent the meaning of the head noun and the meaning of the modifier contribute to the meaning of the compound. If the contribution of the head noun is clear, and the contribution of the modifier is clear but not redundant (or if both the contribution of the head and the modifier are very clear), the compound is judged as relatively plausible. If however the contribution of the modifier is not clear, the similarity between the head and the modifier is used as a back-up resource. In this case, a higher similarity can help to make an otherwise difficult compound more plausible. Another possibility is that a higher similarity generally comes with a higher plausibility, but this positive effect can be “blocked” by redundant modifiers. Furthermore, if this similarity between the modifier and the head is low, the familiarity with the compound can be exploited as an additional back-up plan, with a higher familiarity boosting the perceived plausibility of compounds with dissimilar constituents.

Thus, our results suggests a threefold mechanism, with each step involving different levels of semantic complexity: In the first part, a meaning representation for the whole compound is necessary; the second part operates on the level of single words (the constituents); and the third part operates on the level of familiarity, which does not necessarily imply any representation of meaning. Therefore, in this proposed mechanism, in order to judge the plausibility of compounds humans rely on lower-level information as back-up mechanisms in cases where higher-level information is not clear or redundant.

Importantly, with the account presented here we do not claim that humans go through any of these processes consciously, in that they explicitly judge how well they can relate a *tree house* to a house, or in which way and to what extent the meaning of *tree* contributes to the meaning of *tree house*, in order to produce plausibility judgements. We rather propose, following the outlines of the Plausibility Analysis Model [29], that they initially try to build up a representation for *tree house*, and then judge whether this representation they built makes sense, and how well it fits their prior experience. The measures we proposed, and that we found to predict plausibility judgements, pick out on different aspects of such an alignment: How well does the combined concept fit into the relevant category (does it make sense for some *house* to be a *tree house*, or a *neck house*)?; can the role of the modifier and its contribution to the compound be identified (what exactly is an *eggplant house*, and how does a *roof house* differ from any normal house)?; and can the constituents be aligned (what do *beard* and *house* have in common to be combined into *beard house*)? Therefore, we argue that the measures we propose are aspects of a noun compound that (partly) *constitute* its plausibility. They can be used to describe whether a person is able to arrive at a representation she considers meaningful and plausible, or, possibly more importantly, ways in which she fails to do so, leaving her with a “fuzzy” representation she cannot integrate. Whether humans actually *use* this information in terms of cognitive processes, as in the model proposed in this section, remains subject to further empirical investigations.

Note that our argumentation along the lines of the Plausibility Analysis Model [29] implies a temporal sequence in plausibility judgements, in that we assume that a representation for the compounds is formed before the plausibility of this representation is assessed. This assumption is in itself an empirical hypothesis, which could be tested in studies employing compositional vs. non-compositional items (i.e., semantically transparent vs. opaque items), as well as items with high vs. low plausibility judgements. These could be separate classes of items, or the factors could be crossed in a 2 × 2 design. This material can then be presented to participants in an eye-tracking paradigm [107], with tasks such as just reading the compounds, lexical decision tasks, or timed plausibility judgements. Our hypothesis for such a study would be that differences between compositional vs. non-compositional items should be observed already in the first fixation duration [106], [108], while differences between plausible and non-plausible items should only emerge at later stages, and therefore only at the total gaze duration. A similar approach using timed plausibility judgements could also be implemented in an ERP paradigm, where differences between compositional vs. non-compositional items should be observed at earlier components of the ERP than differences between plausible vs. non-plausible compounds (compare [109] for such an analysis of early vs. late processing differences in ERPs).

#### Explicit Judgements vs. Processing

Note that we do not necessarily assume that these parts have to take place in the sequential (or even hierarchical) order they were described in. In principle, we can also assume that the information is processed in a massively parallel and interacting fashion. In such a model, the different types of relatedness, as well as the information on familiarity, are checked at the same time, and then integrated in a final step to come up with a plausibility judgement. The data we considered includes explicit plausibility judgements, and no timing information. Therefore, while we can make statements about which factors influence those plausibility judgements, we cannot make claims about the temporal order in which these factors are considered, and about the *processing* of noun compounds. Indeed, a parallel explanation of our results would be in line with most recent models of compound processing [110], [111].

In a study on the processing of noun compounds, [112] investigated reaction times in lexical decision tasks from the English Lexicon Project [113]. In that task, participants had to decide whether a compound presented to them existed as a word or not (the compounds where therefore written as one string, for example *swordfish* and not *sword fish*). [112] found that those lexical decision times could be predicted from the relatedess between modifier and compound as well as between head and compound, according to DSMs (the measures we refer to as Modifier Proximity and Head Proximity): they were relatively short if both were high, and longer if any one of them was low. Results in line with these findings were also obtained in [114], wo found that compounds with an transparent head took shorter time to process than compounds with a non-transparent head. This pattern of findings resembles parts of our results in this study, where we find high plausibility ratings for compounds where both Head Proximity and Modifier Proximity are high. However, different to our results, [112] did not find shorter reaction times also for lower Head Proximities if the Modifier Proximity was medium-high.

Thus, we find relatively different results for explicit judgements on the plausibility of noun compounds as compared to the processing of noun compounds. Therefore, we assume that two at least partly different mechanisms are involved in the two different tasks. While it is necessary for explicit judgements on the plausibility to have an interpretable representation of the compound meaning that can be elaborated on, lexical decisions do not depend on such a representation. In the latter, only the existence of the compound as a lexicalized word has to be judged, and not whether it is plausible, which can also be the case for novel compounds. Also, lexical decision usually implies a certain degree of time pressure to the participants; as a consequence, the semantic access in this task may be coarser, with no role to be played by the nuanced interplays emerging in the present study.

#### Compositionality

Crucially, as stated above, we assume that the meanings of compounds are derived *compositionally*, that is, they are constructed from the constituents when a compound is encountered. Our results show that we can predict human plausibility ratings of compounds by applying plausibility measures on distributional vectors that were derived using compositional methods in distributional semantics. Since a large part of the data set we used consisted of non-attested, that is novel noun compounds, we had to derive meaning representations also for those novel compounds. To accomplish this, compositional methods are necessary, since we cannot derive a representation for those compounds on the basis of prior experience with them (i.e., their co-occurrences the corpus). Compositional methods in DSMs provide a powerful way to derive such meaning representations for novel compounds, since these can be obtained for any compound where the constituents are known (more specifically, for the Lexical Function method we applied, the head noun must be known, and the modifier must have been encountered as a modifier in compounds before).

However, the utility of compositional methods in DSMs is not purely practical. Indeed, it has been proposed that these mechanisms may constitute plausible models for meaning combination at the cognitive level. In fact, in the present paper the same compositional method was able to successfully predict plausibility judgements for familiar, attested compounds. That is, our results are based on representations for those familiar compounds that also are obtained compositionally. In that, we are in line with the view that composition (i.e., conceptual combination) always takes place in the interpretation of compounds, for novel compounds as well as for familiar, more lexicalized ones [115], [116], [117]. According to [115], while it is obvious that the meaning of novel compounds has to be derived compositionally, as they cannot be represented in the mental lexicon, there are also reasons to assume that the meaning of familiar compounds is obtained compositionally as well. For example, [116] found that reaction times in sense-nonsense judgement tasks (as well as lexical decision times) for familiar compounds such as *snowball* were faster if they were primed with another compound that used the same relation between modifier and noun (such as *snowfort*, with a MADE OF relation) as opposed to a compound using a different relation (such as *snowshovel*, with a USED FOR relation). The same pattern can be found for novel compounds as target words [53]. These results show that even highly lexicalized compounds such as snowball are not represented as a fixed entry in the mental lexicon, but the relation between the constituents is represented, and therefore the constituents themselves are also represented. This in turn suggests that conceptual combination always is involved in obtaining representations, even for highly lexicalized compounds. More arguments for this claim can be found in [115]. Furthermore, it has been shown that conceptual combination is a very fast process [108], [7]. This suggests that conceptual combination is a rather automated process, which takes place whenever a compound is processed.

#### Implications of the Lexical Function Method

The specific method we applied, and that gave the best initial results, was the Lexical Function method [81], [82], in which the modifier is conceptualized as a function that is applied on the vector representation of the head noun. In that, the modifier and the head play two very different roles in the composition process: The head noun is represented as a vector in the semantic space, with a number of dimensions and numerical values on these dimensions (which can be translated as features, Griffiths et al., 2007, Dinu & Lapata, 2010). Therefore, as in the literature on conceptual combination, the entity described by head noun is represented as a prototype concept. The role of the modifier is then to change these numerical values on the dimensions. More specifically, the matrix representation for the modifier as a Lexical Function gives an exact instruction of to what extent each dimensional value of the head noun vector influences each dimensional value of the compound vector. In that, the modifier guides the composition, in that it specifies how exactly the compound representation is obtained from the head noun representation, given a specific modifier. In more general terms, the head noun provides a structure for the composition process, in terms of a concept representation, and the modifier provides the process to be applied on that structure. This is in line with various views on conceptual combination [42], [52], [15], [16], and can, in our view, also be seen as an abstraction of lexicalist approaches to compounding (e.g., [39], [26]), with regard to the underlying idea that the meaning of a compound is obtained from its constituents, with features of the head noun being changed with respect to the modifier.

On a cognitive level, the Lexical Function composition method can be interpreted in terms of association learning [7]: Since in DSMs vector representations of word meanings are derived through co-occurrence counts, these representations are obtained through learning which words occur together, and which words occur in the same contexts (see also [57]), for example by association. Lexical Functions are learned associations on a higher level: A Lexical Function representation for a given modifier is obtained by learning from compounds including this modifier, and it stores the information on how the (distributional) meanings of the compounds are related to the meanings their respective head nouns. For example, the Lexical Function for *moon* captures the relation between *walk* and *moon walk*, *landing* and *moon landing*, *stone* and *moon stone*, and so on. It has thereby learned the regularities in the relation between a variety of compound meanings and head noun meanings that are specific to the modifier *moon*. When encountering a new (potentially novel) compound, these regularities can be applied to the known head noun meaning in order to obtain a meaning for the compound. As discussed in the introduction, this way of obtaining compound meanings is compatible with the literature on conceptual combination [42], [15, 16]. Furthermore, it specifies the claim of the Concept Specialization Model [46], [47] that background knowledge determined which dimensions of the head noun concept are changed to what degree by the modifier: This background knowledge is obtained by learning from experience how the modifier changes the meaning (i.e., the distributional pattern) of head nouns in compounds that are encountered.

This implies that, for each noun in the vocabulary (at least those that were encountered as modifiers before), there have to be at least two representations in the memory: Once as a single word, and once as a function to be applied in compounding, that is as a compounding “instruction”. However, in that our model is not more complex than for example the Concept Specialization Model [46], [47], where it also has to be stored in the background knowledge which slots of which head nouns are filled by the modifier. For this, relying only on a representation of the modifier as a prototype schema is not sufficient; instead, the knowledge about which modifier typically fills which slots of which kind of head noun has to be stored as well. As argued above, Lexical Functions can be interpreted as a formalization of this knowledge.

Obviously, a potential drawback of the Lexical Function model we employed is that the representation of the modifier itself (i.e., its distributional vector) plays no direct role in the compositional process, as it does not affect the computation of the compound vector, and hence the representation of the combined concept. Therefore, the model implicitly assumes that the modifier concept *in itself* is not employed in conceptual combination. From a psychological perspective, this assumption seems questionable. However, one objection to this point is that it in turn assumes that the modifier lexical functions themselves are completely independent from the modifier concept. Since the lexical functions are estimated from the context in which words appear as modifiers in a compound, this claim is difficult to hold. Especially, as discussed earlier, lexical functions encode the regularities how a modifier typically changes the meaning of head nouns, and this in turn should depend substantially on the modifier concept (however, not as encoded in its distributional vector).

Another argument which can be interpreted in favour of a lexical-function-like approach to the composition of noun compounds has been made in [118], [41]. Booij demonstrates that there are cases where modifiers behave very similar to affixes if they are used within compounds. To put it in the words of [119], “there is so far no semantic theory that allows us to clearly draw the line between compounding and affixation on semantic grounds” (pp. 327–328). One example is the German word *Haupt*, or the Dutch *hoofd* (literally *head*), which in compounds regularly assumes the meaning of *main*: A *Hauptbahnhof* is a *main station*, a *Haupteingang* is a *main entrance*, and so on. Lexemes that occur as real words in the lexicon, but adopt a different meaning when used in compounds, are referred to as *affixoids* or *semi-affixes*. Such examples can also be found in English, see *head office* or *head nurse*. One can, in principle, adopt such an affixoid view of modifiers in compounds in general: According to such a view, the modifier meaning in a compound (or the contribution of the modifier) does not need to be necessarily the same as its meaning when used as a single word. Instead, each word when used as a modifier in principle has the possibility to influence the meaning of a head noun in a very idiosyncratic way. This has to be learned from other contexts in which that word is used as a modifier (compare also [42], [52]). According to such an approach in its generalized form, where modifiers are seen as affixes, the lexical function method is an adequate implementation of modifier contributions to compounds, since it has been shown to capture regular affixation well [7].

This being said, a compositional model that might be psychologically more adequate is the Full Additive model proposed in ([80], see S1 Appendix). In this model, the compound meaning is computed as

c=A·m+B·h (10)

with *c* being the *n*-dimensional compound vector, *m* and *h* being the *n*-dimensional modifier and head vectors, respectively, and *A* and *B* being *n* × *n*-dimensional weight matrices. As with in the Lexical Function method, the weight matrices are estimated from a training set. As can be seen, in the Full Additive model both constituent vectors play a role in the computation of the compound vector, and the weight matrices specify how much each dimension of each constituents contributes to each dimension of the compound. The difference between the Full Additive and the Lexical Function model is that the Lexical Function model assumes this contribution pattern to be idiosyncratic for each modifier word.

The Full Additive model has properties that possibly make it more psychologically adequate. The most important point is that it allows to compute compound meanings including *any* modifier and head word for which a distributional vector is available (i.e., a reader has in her mental lexicon), and it thus far more productive than the Lexical Function model that is restricted to compounds whose modifiers were often encountered as modifiers before. Furthermore, the Full Additive model assumes that the dimensional values of both the modifier and head concept directly influence the compound meaning.

However, in our analysis, we still chose to employ the Lexical Function model as a compositional model. The main reason for doing so was a purely empirical one: This model outperformed the Full Additive model in our initial analysis (see S1 Appendix), in that it predicted the human plausibility ratings far better.

### Conclusion

In the present study, we employed compositional methods in distributional semantics to predict human plausibility ratings for noun compounds. These methods can be seen as a formalized implementations of conceptual combination. This formalization allows us to conduct fine-grained analyses of which factors influence the plausibility of compounds.

In this analysis, we found that different types of semantic *relatedness* concerning a compound help us in understanding plausibility: The relatedness between the compound and the head, the relatedness between the compound and the modifier, as well as the similarity between the head and the modifier. Those variables interact with each other. A higher relatedness between head and compound is associated with higher plausibility, but more so if modifier and compound are optimally related. The similarity between the constituents is associated with a slightly higher plausibility, but only if the relation between modifier and compound is not too redundant. Furthermore, the familiarity with the compound has a stronger effect the lower the similarity between the constituents is.

The relations between the constituent meanings and the compound meaning can be seen as the contributions of the former to the compound meaning. The identification and assessment of these contributions seem to play a major role for plausibility judgements, which was already found in other studies [7], [31]. Investigating those complex interactions between different semantic variables requires an exact model of how the meaning of a compound is derived from its constituents. Compositional methods in distributional semantics provide such a model, helping us to understand which semantic variables influence the plausibility of compounds, and therefore what leads some compounds to be perceived as more plausible than others.

## Supporting Information

S1 Appendix Employed Composition Methods. A pre-test comparing different composition methods.
(PDF)

S2 Appendix Modelling the data. A detailed description of the GAM data analysis.
(PDF)

S3 Appendix Data Set. The main data set used for the analysis in this study, including the relevant plausibility measures.
(TXT)

S4 Appendix Training Set. The training set used to derive the Modifier Lexical functions.
(TXT)
