# Supplementary material for: Understanding Karma Police: The Perceived Plausibility of Noun Compounds as Predicted by Distributional Models of Semantic Representation
Source: PLoS One. 2016 Oct 12;11(10):e0163200. doi: 10.1371/journal.pone.0163200 (PMC5061382; doi:10.1371/journal.pone.0163200)
Supplement: S1 Appendix — A pre-test comparing different composition methods. (PDF) [file pone.0163200.s001.pdf]

S1 Appendix: Employed Composition Methods

We employed the following composition methods proposed by Mitchell and Lapata (2010) to compute the vector for a compound $c = (c_1, ..., c_i, ..., c_n)$ from the vector of its modifier noun $m = (m_1, ..., m_i, ..., m_n)$ and its head noun $h = (h_1, ..., h_i, ..., h_n)$:

- **Additive Model:** $c = m + h$
- **Weighted Additive Model:** $c = a \cdot m + b \cdot h$, with a and b being scalars
- **Multiplicative Model:** $c_i = m_i + h_i$
- **Dilation:** $c = (m \cdot m) \cdot h + (\lambda - 1) \cdot (m \cdot h) \cdot m$, λ being a scalar
- **Circular Convolution:** $c_i = \sum_j m_j \cdot h_{i-j}$, subscripts are interpreted modulo n

Furthermore, we employed the following regression-based trained models:

- **Full Additive:** $c = A \cdot m + B \cdot h$, with A and B being $n \times n$ matrices
- **Modifier Lexical Function:** $c = M \cdot h$, with M being an $n \times (n + 1)$ matrix
- **Head Lexical Function:** $c = H \cdot m$, with H being an $n \times (n + 1)$ matrix

The Full Additive Model (Guevara, 2010) is a generalization of the Weighted Additive Model. Here, modifier matrices are estimated for specific syntactic roles from a training set. To estimate A and B for noun compounds, for example, one computes the observed phrase vectors for all noun compounds in the source corpus, and then estimates A and B by solving a regression problem, as described for the Lexical Function approach in the main article.

The Head Lexical Function model is closely related to the Modifier Lexical Function Model, except the modifier is seen as the vector whose values are modified by the head noun it is combined with. The matrix for the head noun *house*, for example, is obtained by

collecting observed vectors for compounds such as *boat house*, *tree house*, and *country house*, from which the matrix is estimated by solving a regression problem.

Note that we did not employ a whole-word approach, where we just took the observed phrase vectors as vector representations for the compounds. This is caused by the fact that a large proportion of the data set we analysed consisted of non-attested compounds for which such vector are not available, since the compounds do not appear (or are extremely rare) in our source corpus.

Parametrization

While some of the aforementioned composition methods are parameter-free (namely, the Additive, Multiplicative, and Circular Convolution method), the other ones rely on setting parameter values to compute the phrase vectors.

For the Weighted Additive and the Dilation model, we obtained one set of parameters by taking the parameters that Mitchell and Lapata (2010) found to give the best results for noun compounds over a variety of tasks. Those parameter values are displayed in Table 1.

The training set for the Modifier Lexical Function is described in the main article. The training set for the Head Lexical Function consisted of all the noun pairs in the corpus (a) where the *second* noun appeared as a constituent in the item set, and (b) that occurred at least 20 times in the corpus. No lexical function was estimated for head nouns with less than 50 training examples. For this reason, we excluded 186 head nouns from the training set. In total, we collected 31,518 training word pairs for 205 different Head Lexical Functions (up to 1,615 training items per head noun). Since we eliminated 186 modifiers from the data set, we obtained 1,642 compound vectors (773 for attested and 869 for unattested compounds).

The training set for the Full Additive Model was obtained by pooling the two training sets for the Modifier Lexical Function and the Head Lexical Function.

Finally, the same procedure of parameter estimation by training can not only be applied

for the Full Additive or the Lexical Function model, but also for the Weighted Additive and the Dilation model. We used the same training set as for the Full Additive model to estimate the parameters for these models, which are displayed in Table 1. These trainings were conducted using the free software toolkit DISSECT (Dinu, Pham & Baroni, 2013).

Table 1

Parameter values chosen for the parameterized composition methods. (ML) refers to values obtained form Mitchell and Lapata (2010), (T) refers to the parameter values obtained through training

Model	Parameter values
Weighted Additive (ML)	$a = 0.32$, $b = 0.68$
Weighted Additive (T)	$a = 0.48$, $b = 0.52$
Dilation (ML)	$\lambda = 8.3$
Dilation (T)	$\lambda = 3.9$

Initial Comparison of Composition Methods

For our initial method comparisons, we computed Spearman rank-correlations between the plausibility measures and the mean plausibility ratings for each employed composition method. These correlation are displayed in Table 2. Constituent Similarity is not considered here, since it is not dependent on the composition method that is employed and therefore the same for all methods.

These results suggest that the Modifier Lexical Function performs particularly well in combination with most of the plausibility measures. To get an overview over the overall performance of the different methods, we further conducted simple linear regression analyses of the following type:

$$\text{rating} \sim \text{neighbourhood\_density} + \text{modifier\_proximity} + \text{head\_proximity} + \text{entropy}$$

Table 2

Spearman Correlations between semantic transparency measures and mean plausibilities per item. Significance levels are coded by: * $p < .05$, ** $p < .01$, *** $p < .001$

Composition Method	Neighbourhood Density	Head Proximity	Modifier Proximity	Entropy
Additive	.166***	.054*	.225***	-.058**
Multiplicative	.111***	.160***	.187***	.208***
Weighted Additive (ML)	.004	.008	.240***	-.058**
Weighted Additive (T)	.105***	.041	.231***	-.035
Dilation (ML)	.089**	-.169***	.214***	.197***
Dilation (T)	-.016	-.205***	.214***	.180***
Circular Convolution	.031	-.023	-.020	.000
Full Additive	.083***	-.011	.155***	.222***
Modifier Lexical Function	.303***	.441***	.087***	-.280***
Head Lexical Function	-.075**	.011	-.013	-.021

Table 3

Coefficients of determination by composition method for the simple linear regression model

Composition Method	r^2
Additive	.05
Multiplicative	.04
Weighted Additive (ML)	.05
Weighted Additive (T)	.05
Dilation (ML)	.04
Dilation (T)	.04
Circular Convolution	.002
Full Additive	.07
**Modifier Lexical Function**	**.21**
Head Lexical Function	.02

Table 3 shows the coefficients of determination (r^2) for each composition method. Because the Modifier Lexical Function clearly emerged as the highest-performing composition method in these initial analyses, we decided to focus on this method in our actual analysis of the plausibility ratings. Note that the Modifier Lexical Function is also in line with the psychological theories on conceptual combination, which is a further advantage of choosing this method (see the *Discussion* in the main article, as well as Marelli & Baroni, 2015).
