# Supplementary material for: Understanding Karma Police: The Perceived Plausibility of Noun Compounds as Predicted by Distributional Models of Semantic Representation
Source: PLoS One. 2016 Oct 12;11(10):e0163200. doi: 10.1371/journal.pone.0163200 (PMC5061382; doi:10.1371/journal.pone.0163200)
Supplement: S2 Appendix — A detailed description of the GAM data analysis. (PDF) [file pone.0163200.s002.pdf]

S2 Appendix: Modelling the data

Baseline Model. The analyses reported here were conducted using the package *mgcv* (Wood, 2015) in R (R Core Team, 2015). In a first step, we identified a baseline model containing fixed effects for the covariates, as well as random effects for head nouns and modifier nouns. We started from the following model containing all those effects:

$$\begin{aligned}\text{rating} \sim\ & \text{length(modifier)} + \text{length(head)} + \\ & + \text{log(frequency(modifier))} + \text{log(frequency(head))} \\ & + \text{log(frequency(bigram))} + \text{log(frequency(reversed bigram))} + \text{PMI} \\ & + \text{s(modifier,bs="re")} + \text{s(head,bs="re")}\end{aligned}$$

After removing non-significant predictors from the model, we obtained the following baseline model:

$$\begin{aligned}\text{rating} \sim\ & \text{length(modifier)} + \text{log(frequency(bigram))} + \text{log(frequency(reversed bigram))} + \text{PMI} \\ & + \text{s(modifier,bs="re")} + \text{s(head,bs="re")}\end{aligned}$$

Adding Plausibility Measures. Starting from the baseline model, we tested for effects of the plausibility measures in a step-wise procedure, using Likelihood-ratio tests (implemented in the `anova()` command in R). The procedure we applied consisted of the following steps:

- (A) Include an additional linear or non-linear parameter in the model for any semantic transparency measure that is not yet part of the model (resulting in 2*[*number of remaining parameters*] rival models). From the set of those rival models where the additional effect significantly improves the model, pick the one with the minimum Akaike Information Criterion (AIC).

- (B) Test whether including any (non-linear) interaction between plausibility measures present in the current model significantly improves the model. If there are multiple such cases, pick the model with the minimum AIC and repeat step (B).

- (C) Go back to step (A). Repeat until no further (linear or non-linear) effect for the semantic transparency measures significantly improves the model.

Table 1 shows the order in which the effects were added to the model. As can be seen, the effect of Modifier Proximity was not significant in itself. However, considering the relatively low *p*-value associated to the effect, we decided to further test for possible interactions including it, which turned out to considerably improve the model.

Influences of compound familiarity. An important feature of the analysed data set is that it consists of attested noun compounds as well as reversed-ordered ones. Therefore, the compounds differ greatly in frequency (and thereby compound familiarity). However, we opted not to include a factor specifying whether a compound was attested or reversed-ordered in our model. Instead, we took the (logarithmic) frequency of the modifier-head bigram as a variable measuring familiarity, since it is far more fine-grained and not artificially dichotimized.

To test for influences of familiarity, we tested whether we can improve the predictions of our current model by including interactions of the semantic transparency measures in the model (and their interactions) with modifier-head pair frequency. The model is significantly improved by including a non-linear interaction between Constituent Similarity and modifier-head pair frequency (see Table 1).

Table 1

Effects added to the model, in the order of their inclusion. s() *indicates non-linear (smoothed) effects, and* te() *indicates non-linear (tensor-product) interactions. The reported test statistics refer to the Likelihood-ratio test comparing a model with and without the respective effect. AIC refers to the AIC of the model including the respective effect.*

Parameter	Δdf (approx.)	$\Delta Deviance$	p	AIC
baseline				4181.88
s(head proximity)	4.87	89.87	<.001	3991.07
s(constituent similarity)	2.33	7.36	.001	3978.20
s(modifier proximity)	2.48	2.83	.095	3976.38
te(modifier proximity,constituent similarity)	0.61	2.85	.008	3970.74
te(head proximity,modifier proximity)	5.28	7.27	.017	3963.65
te(constituent similarity,log(frequency(bigram)))	8.52	18.36	<.001	3935.35

Including any further interactions does not significantly improve the resulting model. Therefore, we end up with the following final model:

$$
\begin{aligned}
\text{rating} \sim\ & \text{te(head proximity, modifier proximity)} \\
& + \text{te(modifier proximity, constituent similarity)} \\
& + \text{te(constituent similarity, log(frequency bigram))} \\
& + \text{length(modifier)} + \text{log(frequency(bigram))} \\
& + \text{log(frequency(reversed bigram))} + \text{PMI} \\
& + \text{s(modifier,bs="re")} + \text{s(head,bs="re")}
\end{aligned}
$$

As confirmed in a counter-check with Wald tests, each parameter term in the final model is indeed significant.
